# Low frequency lattice mode dynamics of cyclotrimethylene trinitramine (RDX) crystal studied by femtosecond time-resolved impulsive stimulated Raman scattering

**DOI:** 10.1038/s41598-023-29179-7

**Published:** 2023-02-13

**Authors:** Guoyang Yu, Yunfei Song, Gangbei Zhu, Zhaoyang Zheng, Qiang Wu, Yanqiang Yang

**Affiliations:** National Key Laboratory of Shock Wave and Detonation Physics, Institute of Fluid Physics, China Academy of Engineering Physics, Mianyang, 621900 People’s Republic of China

**Keywords:** Chemistry, Optics and photonics, Physics

## Abstract

The femtosecond time-resolved impulsive stimulated Raman scattering (fs-ISRS) has been performed to study the low frequency lattice mode dynamics of the RDX crystal. Through Fourier filtering, four lattice mode dynamics is distinguished from the time-resolved spectrum. And the wavenumbers and time constants of these four lattice modes are determined by fitting their dynamic curves. The energy dispersion paths of these four lattice modes are deduced from these fitting parameters. Compared with the other three lattice modes, the lattice mode with wavenumber 30 cm^−1^ has a very longer life time. We consider that the excitation of this lattice mode more likely to cause the damage of the intermolecular interaction under the strong external stimulation.

## Introduction

Lattice modes will be excited firstly when energetic materials are influenced by external stimuluses such as shock waves, impacts, heating, etc. And these excitations of lattice modes make energy of external stimuluses transfer to the internal of energetic materials. Generally, the energy carried by lattice modes has two transfer paths. The one path called multi-phonon up pumping is that the energy will transfer from lattice modes to vibrational modes with higher frequencies through the anharmonic effect^1,2^. The other path is that the energy will transfer between lattice modes and finally relax to the thermal bath. These two energy transfers are closely related to the initial reaction process of energetic materials. Thus, the study of lattice mode dynamics will provide an insight into the micro-mechanism concerning the detonation performance and safety of energy materials.

Compared with vibrational modes in molecules, the frequency of lattice modes of energetic materials is relatively low, usually in the range from dozens of gigahertz to several terahertz. And the energy transfer concerning lattice modes of energetic materials is an ultrafast process, the time scale is usually in the range from hundreds of femtoseconds to several picoseconds. Thus, the measurement of lattice mode dynamics is not easy. For investigating this process, based on its frequency and temporal characteristics, we suggest that the femtosecond time-resolved impulsive stimulated Raman scattering (fs-ISRS) will be an effective method to observe lattice mode dynamics of energetic materials.

In the ISRS method, the ultrashort excitation pulse is used to suddenly drive coherent vibrational motion in the ground electronic state of the molecule. This vibrational motion could be an acoustic vibration in the liquid material, an optic phonon in the crystal material, a molecular vibration, etc. And therefore, this method has been widely applied to investigate vibrational dynamics in liquid materials^3–6^, inorganic crystal materials^7–10^, and organic crystal material^11,12^.

In our work, fs-ISRS has been performed to observe low frequency lattice modes dynamics of the energetic material and the cyclotrimethylene trinitramine (RDX) crystal has been chosen as a sample. The frequencies and life times of four lattice modes can be determined and the dissipation path of energy can be deduced from the experimental data.

## Experiment

**Figure 1:**
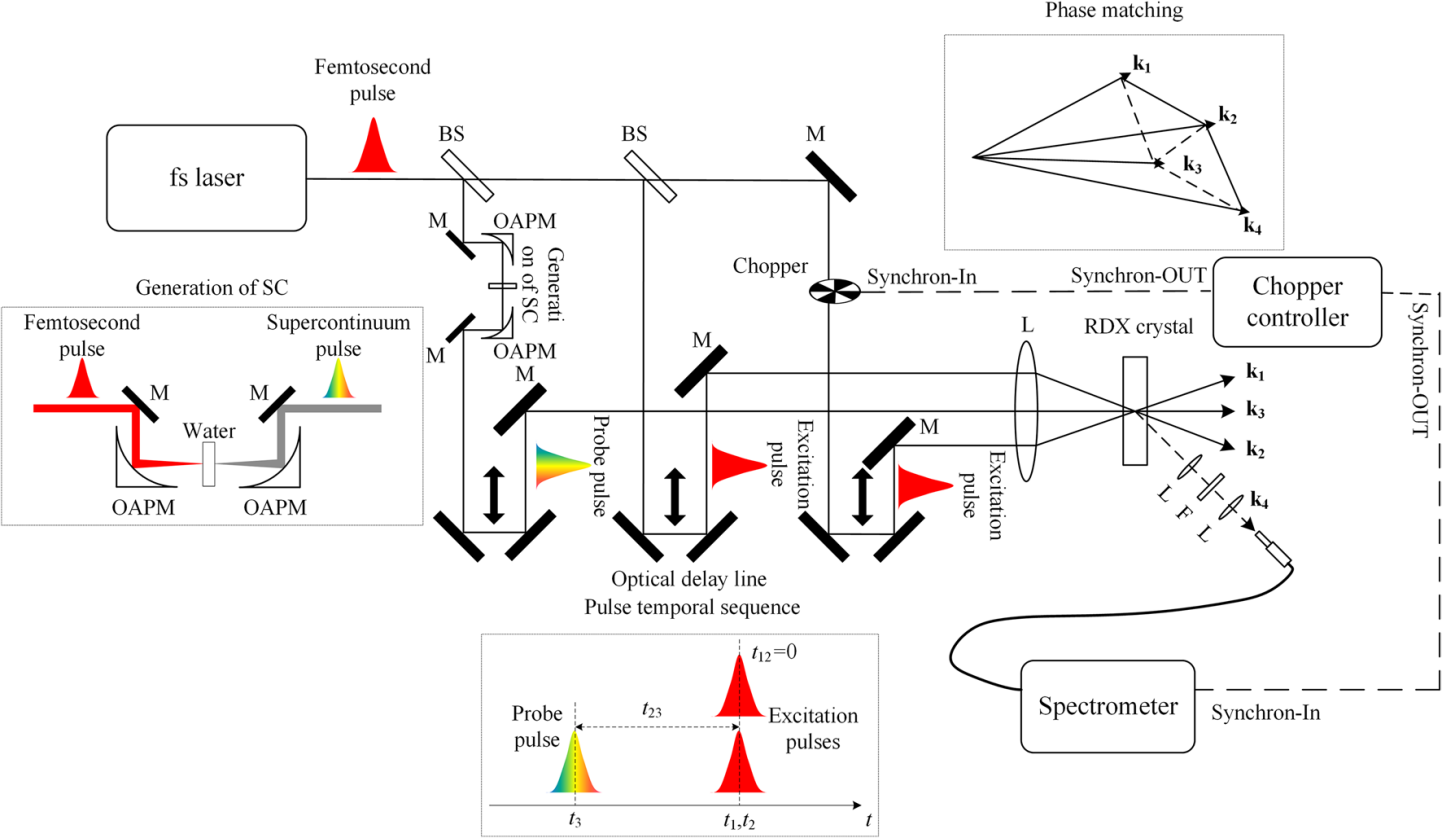
Schematic diagram of the experimental setup. *BS* beam splitter, *M* mirror, *OAPM* off-axis parabolic mirror, *L* lens, *F* filter.

The transient grating geometry was employed in our fs-ISRS experiment and the experimental setup is shown in Fig. 1. A femtosecond pulse (130 fs, 800 nm, 1 kHz) emitted from a Ti:sapphire regeneration amplified laser system (Spitfire, Spectra-Physics) was split into three by two beam splitter. Two of them with the wave vectors **k**_**1**_ and **k**_**2**_ acted as excitation light and their energy-per-pulse was both attenuated to about 2 μJ. The third one which its energy-per-pulse was attenuated to about 1 μJ was focused into the distilled water by an off-axis parabolic mirror to generate the supercontinuum (SC) that acted as probe light with the wave vector **k**_**3**_. The three beams were spatially focused into the RDX crystal in the folded box geometry. The pulse temporal sequence was adjusted by optical delay lines. The interval (*t*_12_) between two excitation pulses was set to zero, and the interval (*t*_23_) between the excitation and probe pulses was scanned. And the time when the excitation and the probe pulses simultaneously arrived at the sample was defined as the zero time. The signal along the phase-matching direction** k**_**4**_ = **k**_**1**_** − k**_**2**_ + **k**_**3**_ was captured by a spectrometer (ULS350, Aventis). A chopper (MC2000, Thorlabs) was placed in the path of one of the excitation beams. And a chopper controller was used to control the phase of the chopper; meanwhile, it outputted a synchronizing signal to the spectrometer. Therefore, if the excitation pulse passes the chopper, the spectrometer can capture a signal. If not, the spectrometer can capture a background. Finally, the signal taken out the background to obtain the pure ISRS signal.

The RDX crystal sample used in this experiment was prepared by solvent crystallization in our laboratory. The more details in the sample preparation have been described in our previous work^13^.

## Results and discussion

**Figure 2:**
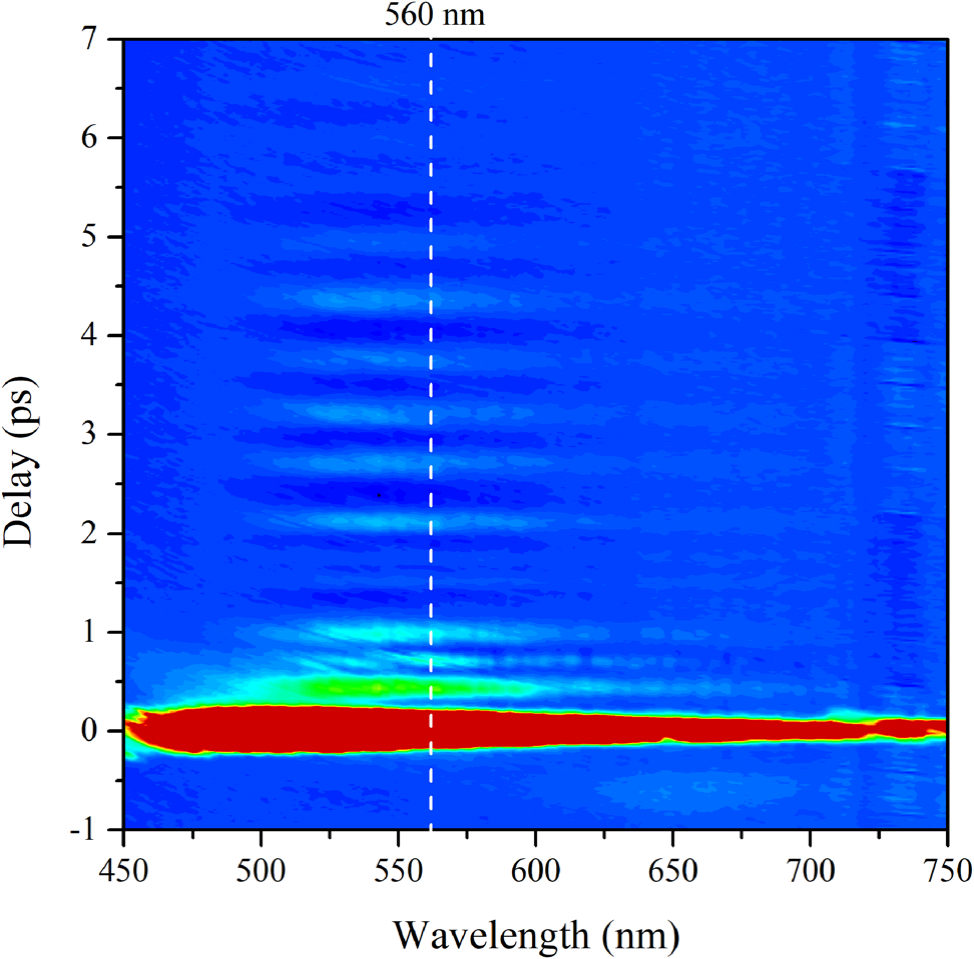
Time- and frequency-resolved ISRS spectrum of RDX shown by the contour map.

The chirp of SC acted as the probe pulse in the experiment caused the dispersion of the ISRS signal. Therefore, the dispersion correction has been performed for the ISRS signal (see the Supporting Information, section “Introduction”). The time- and frequency-resolved ISRS spectrum of RDX is shown by a contour map. As shown in Fig. 2, in the frequency domain, the center wavelength of the ISRS spectrum is about 560 nm which is related to the wavelength of the excitation light, the angle of two excitation lights and the incident angle of the probe light (see the Supporting Information, section “Experiment”). And, in the temporal domain, the ISRS spectrum has a very strong non-resonant electronic background at around the zero time and a complicated oscillation structure after the zero time.

**Figure 3:**
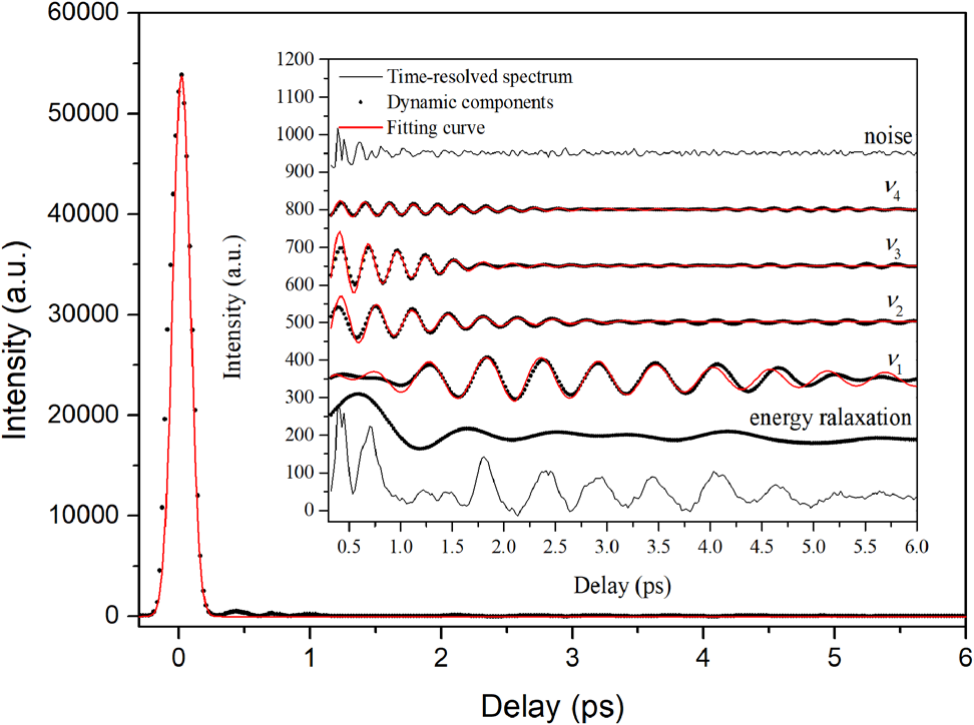
Time-resolved ISRS spectrum at 560 nm. (Inset) Oscillation structures in the time-resolved ISRS spectrum. The vibrational modes are named ν_n_ (n = 1, 2, 3, 4).

The time-resolved ISRS spectrum at 560 nm is taken as an example to describe the general case of the oscillation structure because time-resolved ISRS spectra have similar behaviors at other wavelengths. As shown in Fig. 3, a very strong peak fitted with the Gaussian curve near zero time is the nonresonant electronic background that is related to the electron response to the electric field component of light. As shown in the inset of Fig. 3, three types of dynamic components after the nonresonant electronic background are distinguished through Fourier filtering (see the Supporting Information, section “Results and discussion”). The high frequency component is irregular, which originates from noise. The low frequency component mainly shows an exponential decay which roots in the energy relaxation. The intermediate frequency component are four damped oscillations which results from the vibrational mode dynamics.

The wavenumbers and time constants of the vibrational modes are determined by fitting the damped oscillation curves. The vibrational mode ν1 with wavenumber of 30 cm^−1^ has a rising time of 1.3 ps and a decay time of 5.6 ps. Compared with the vibrational mode* ν*_1_, the vibrational mode *ν*_2_, *ν*_3_ and *ν*_4_ only have decay times of 1.7 ps, 1.3 ps and 2.5 ps. And their wavenumbers are 49 cm^−1^, 60 cm^−1^ and 71 cm^−1^, respectively. According to previous works^14,15^, the vibrational modes *ν*_1_, *ν*_2_, *ν*_3_ and *ν*_4_ are all corresponding to the lattice modes of the RDX crystal, which are mainly related to the intermolecular interaction in the crystal.

**Figure 4:**
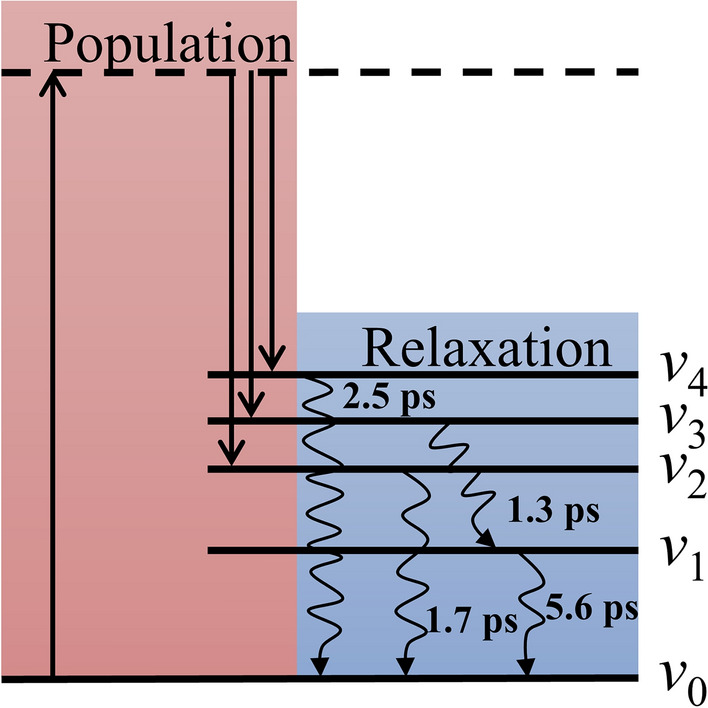
Energy level diagram describing the lattice mode dynamics of the RDX crystal.

According to the fitting information from the time-resolved ISRS spectrum, the dynamics of these lattice modes are described by an energy level diagram as shown in Fig. 4. In the population, the excitation pulses will excite three lattice modes* ν*_2_, *ν*_3_ and* ν*_4_ with wavenumbers of 49 cm^−1^, 60 cm^−1^ and 71 cm^−1^. And the lattice mode *ν*_1_ with wavenumber of 30 cm^−1^ is not excited directly by the excitation pulses because its dynamic curve did not appear at first of the time-resolved ISRS spectrum and has a rising component. In the relaxation, the lattice modes *ν*_2_ and *ν*_4_ exchange energy with the thermal bath directly and their lifetimes are 1.7 ps and 2.5 ps, respectively. And the energy of the lattice mode *ν*_3_ with the wavenumber of 60 cm^−1^ do not relax to the thermal bath and transfer to the lattice mode *ν*_1_ with the wavenumber of 30 cm^−1^. Because the decay component of the dynamic curve of the lattice mode *ν*_3_ matches with the rising component of the dynamic curve of the lattice mode *ν*_1_, and their time constants both are 1.3 ps. And the value of the wavenumber of the lattice mode *ν*_3_ is double compared with the lattice mode *ν*_1_, which makes resonance between these two lattice modes, easily. At last, the lattice mode *ν*_1_ exchanges energy with the thermal bath and its lifetime is 5.6 ps.

The four lattice modes distinguished from ISRS signal are corresponding to the lattice vibration or the global motion of the molecule, which is closely related to the intermolecular interaction such as hydrogen bond and Vander Waals force. Thus, the intermolecular interaction could be damaged when this type of lattice mode is excited under the strong external stimulation. And the longer the lifetime of the lattice mode, the higher the possibility of this damage happens because the energy will stay longer on the lattice mode with a longer lifetime. This damage of the intermolecular interaction shows the plastic dissipation on the macro level such as crack initiation and grain boundary sliding of the crystal, which will significantly influence the subsequent reaction. Compared other three lattice modes, the lattice mode *ν*_1_ has a longer lifetime, which means that the excitation of this lattice mode more likely to cause the damage of the intermolecular interaction under the strong external stimulation.

## Summary

In summary, fs-ISRS has been used to investigate low frequency lattice modes dynamics of the RDX crystal. Four lattice modes named* ν*_1_, *ν*_2_, *ν*_3_ and *ν*_4_ are observed from the time-resolved ISRS spectrum through Fourier filtering, and their wavenumbers and time constants are determined by fitting the dynamic curves. The energy dissipation paths of these four lattice modes have been deduced from the fitting information. In addition, the lattice mode *ν*_1_ has a longer lifetime compared other three lattice modes. We suggest that the excitation of this lattice mode more likely to cause the damage of the intermolecular interaction under the strong external stimulation. In the future work, the laser pulse with the shorter pulse width will be used in this experiment so that the lattice modes with higher frequency will be observed in experimental results. And the multi-phonon up-pumping of energetic materials will be investigated by this fs-ISRS.

## Supplementary Information

Supplementary Information.

## Data Availability

The datasets generated during and/or analyzed during the current study are available from the corresponding author on reasonable request.
