# Supplementary material for: Low frequency lattice mode dynamics of cyclotrimethylene trinitramine (RDX) crystal studied by femtosecond time-resolved impulsive stimulated Raman scattering
Source: Sci Rep. 2023 Feb 13;13:2530. doi: 10.1038/s41598-023-29179-7 (PMC9925810; doi:10.1038/s41598-023-29179-7)
Supplement: Supplementary file 1 — Supplementary Information. [file 41598_2023_29179_MOESM1_ESM.docx]

**Supporting Information**

**Low Frequency Lattice Mode Dynamics of Cyclotrimethylene Trinitramine (RDX) Crystal Studied by Femtosecond-Resolved Impulsive Stimulated Raman Scattering**

*Guoyang Yu, Yunfei Song, Gangbei Zhu, Zhaoyang Zheng, Qiang Wu, Yanqiang Yang*

**5 pages**

**1. Dispersion correction**

The chirp of supercontinuum (SC) acted as the probe pulse in the experiment caused the dispersion of the ISRS signal as shown in Figure S1(a). The dispersion correction curve is obtained by finding the maximum value of the intensity at every column along the x (wavelength) axis, which is fitted by Cauchy dispersion formula (*n* = a/*λ*^4^+b/*λ*^2^+c, *n* and *λ* denote the refractive index and the wavelength) as shown in Figure S1(b). Therefore, as show in Figure S1(c), the dispersion of the ISRS signal can be corrected according to the dispersion correction curve.


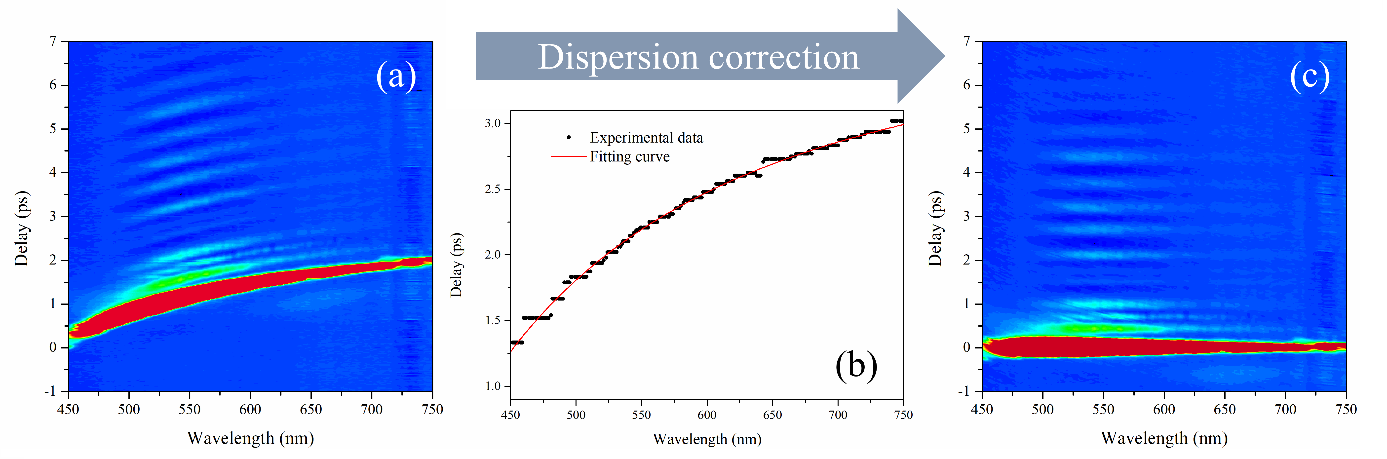


Figure S1. The contour map of ISRS signal of RDX crystal before (a) and after (c) dispersion correction, (b) The dispersion correction curve

**2. Center wavelength of the ISRS spectrum**

The geometry of three beams in the ISRS experiment is shown in Figure S2. The plane in which the lens lies and the intersecting plane of the three beams are defined as (*x*′, *y*′) and (*x*, *y*), respectively. The vertical direction of these two planes is defined as *z* axis. The angle between the excitation beam and *z* axis is defined as *α* and the angle between the projection of the probe beam in the plane (*x*, *z*) and *z* axis is defined as *θ*.


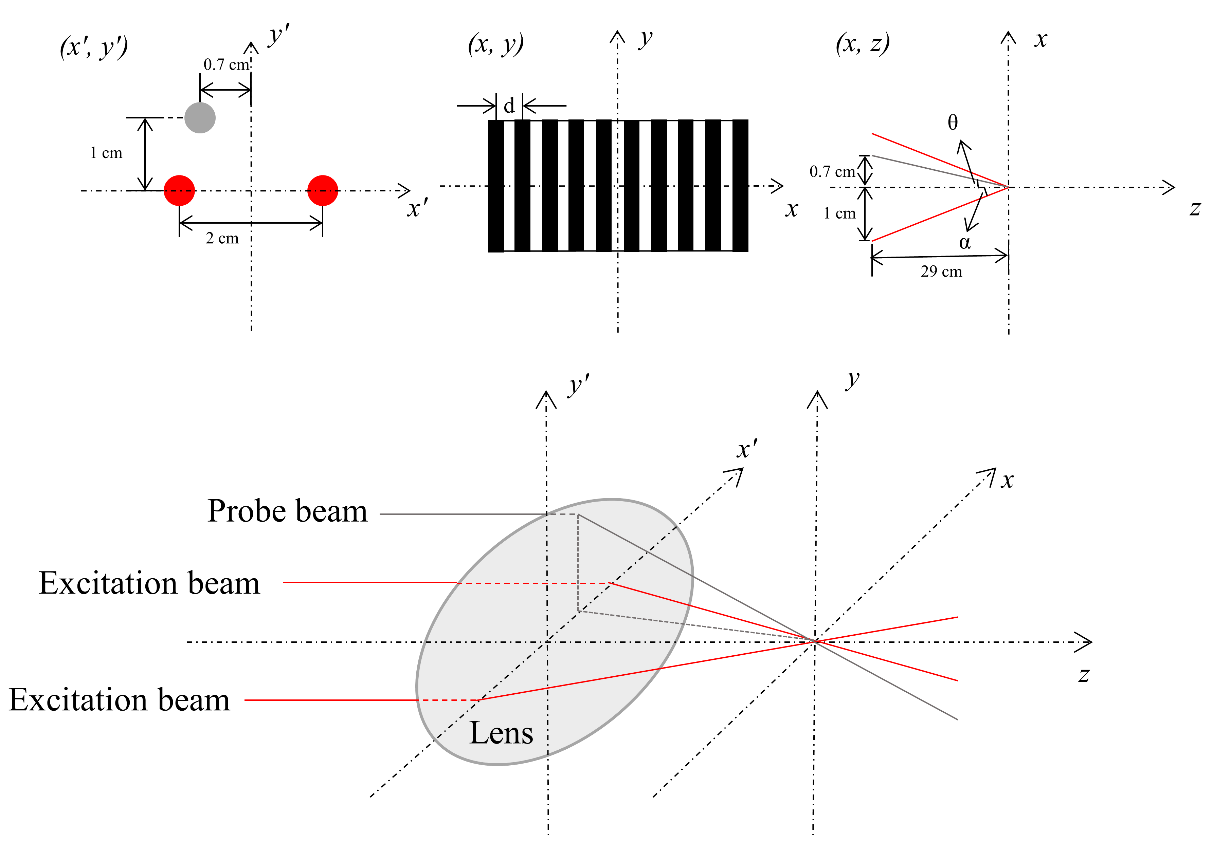


Figure S2. The geometry of three beams in the ISRS experiment

Two intersectant excitation beams generate a serious of volume gratings in the RDX crystal around the plane (*x*, *y*). The grating constant can be express as

$d=\lambda/{\left[ \cos\left( 90^{\circ}-\alpha\right)-\sin\left( 90^{\circ}+\alpha\right) \right]=\lambda/{2\sin\alpha}}$ (2.1)

where *λ* denotes the wavelength of the excitation light. And the diffraction of the probe light is determined by Bragg condition.

$2d\sin\theta=k\lambda'$ (2.2)

where *λ*′ denotes the wavelength of the diffraction signal. Therefore, the relationship between the wavelengths of the excitation light and the diffraction signal can be built by simultaneous Eq. (2.1) and (2.2).

$\lambda'=\lambda\frac{\sin\theta}{\sin\alpha}$ (2.3)

In plane (*x*′, *y*′), the coordinates of two excitation beams and the probe beam are (-1, 0), (1, 0), and (0.7, 1) respectively so that the trigonometric values of the angles *α* and *θ* can be calculated according to locations of three beams and the distance between the lens and the cross point of the three beams. The tangent values of the angles *α* and *θ* are approximately equal to the sinusoidal values of them because the distance between the lens and the cross point of the three beams is much larger than the distances of the excitation and probe beams to the y′ axis. According to Eq. (2.3), the value of the diffraction signal wavelength is approximately equal to 0.7 times of the value of the excitation light. Therefore, the center wavelength of the ISRS spectrum is about 560 nm when the center wavelength of the excitation light is 800 nm.

**3.** **Fourier filtering**

In ISRS spectroscopy, two excitation pulses form a standing wave to cause forced vibrations for phonon mode. For phonon mode *α*, the equation of motion is

$\rho_{\alpha}\left( \frac{\partial^{2}Q^{\left( \alpha\right)}}{\partial t^{2}}+2\gamma_{\alpha}\frac{\partial Q^{\left( \alpha\right)}}{\partial t}+\omega_{\alpha0}^{2}Q^{\left( \alpha\right)} \right)=\sum_{ij} \left( \frac{\partial\varepsilon_{ij}}{\partial Q^{\left( \alpha\right)}} \right)_{0}F_{ij}$ (3.1)

where $\rho_{\alpha}$ is the corresponding inertia density, $\omega_{\alpha0}$ is the natural frequency, and $\gamma_{\alpha}$ is a phenomenological damping constant. It is assumed that the mode is nondispersive. $Q^{\left( \alpha\right)}$ and $F_{ij}$ can be in either *q* space or *r* space. Thus, the corresponding Green’s function is given by

$\rho_{\alpha}\left( \frac{\partial^{2}}{\partial t^{2}}+2\gamma_{\alpha}\frac{\partial}{\partial t}+\omega_{\alpha0}^{2} \right)G^{\left( \alpha\right)}\left( t \right)=\delta\left( t \right)$ (3.2)

The solution for underdamped modes ($\omega_{\alpha0}^{2}-\gamma_{\alpha}^{2}\equiv\omega_{\alpha}^{2}>0$) is

$G^{\left( \alpha\right)}\left( t>0 \right)=\frac{e^{-\gamma_{\alpha}t}\sin\left( \omega_{\alpha}t \right)}{\rho_{\alpha}\omega_{\alpha}}$ (3.3)

The time-dependent ISRS signal is given by

$I\left( \boldsymbol{q},t \right)\propto\left| G^{\left( \alpha\right)}\left( \boldsymbol{q},t \right) \right|^{2}=A\left[ \frac{e^{-\gamma_{\alpha}t}\sin\left( \omega_{\alpha}t \right)}{\rho_{\alpha}\omega_{\alpha}} \right]^{2}$ (3.4)

According to double Angle formula of trigonometric function, the Eq. (3.4) can be transformed into

$I\left( t \right)=A\exp\left( -2\gamma_{\alpha}t \right)+A\exp\left( -2\gamma_{\alpha}t \right)\cos\left( 2\omega_{\alpha}t+\varphi\right)$ (3.5)

For multiple modes, the time-dependent ISRS signal can be rewritten to

$I\left( t \right)=\sum_{i} A_{i}\exp\left( -2\gamma_{i}t \right)+\sum_{i} A_{i}\exp\left( -2\gamma_{i}t \right)\cos\left( 2\omega_{i}t+\varphi_{i} \right)$ (3.6)

where $\gamma_{i}$ and $\omega_{i}$ are the relaxation rate and the circular frequency of mode *i* respectively.

Obviously, it is difficult to directly fit a complex ISRS signal containing multiple modes by using Eq. (3.6). The Fourier filtering can effectively solve this problem. At first, Fourier transform is performed for the whole ISRS signal to determine frequency components. And then, each frequency component can be distinguished through Fourier filtering. The low frequency component mainly corresponds to the first term in Eq. (3.6) and can be expressed as

$I_{\mathrm{ER}}\left( t \right)=\sum_{i} A_{i}\exp\left( -\frac{2t}{\tau} \right)$ (3.7)

where $\tau$ is the life time of mode *i*, which is inverse of $\gamma_{i}$. According to Eq. (3.6), the low frequency component shows multiple exponential decay, which is related to the energy relaxation of multiple modes. According to Eq. (3.6) and (3.7), for each mode, the dynamics can be expressed as

$I_{\mathrm{OS}}\left( t \right)=Ae^{-{2t}/\tau}\cos\left( 2\omega+\varphi\right)+B$ (3.8)

where *A, τ, ω, φ* and *B* denote the amplitude, the life time, the circular frequency, the phase, and the intensity of the background, respectively. And the variable *t* denotes the delay time.


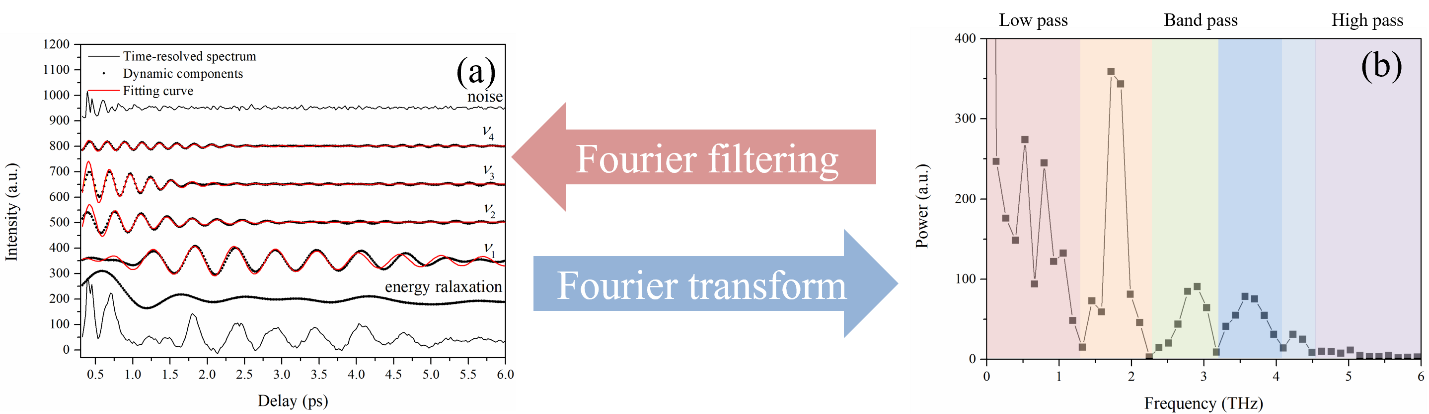


Figure S3. (a) Oscillation structures in the time-resolved ISRS spectrum (b) Fourier transform power spectrum

As shown in Figure S3(a), the time-resolved ISRS spectrum has a complex oscillation structure so that the direct fitting with Eq. (3.6) for this spectrum is very difficult. The Fourier filtering has been performed to distinguish dynamic components in this time-resolved ISRS spectrum. At first, the power spectrum is calculated through Fourier transform as shown in Figure S3(b). Six frequency regions are divided according to the spectral structure of Fourier transform power spectrum. The low pass and the high pass Fourier filtering are applied to the low frequency and the high frequency regions respectively, and the band pass Fourier filtering are applied to four intermediate frequency regions. As show in Figure S3(a), three types of dynamic components are distinguished through Fourier filtering. The high frequency component is irregular, which originates from noise. The low frequency component mainly shows multiple exponential decay which roots in the energy relaxation. The intermediate frequency component are four damped oscillations which results from the vibrational mode dynamics. Therefore, the vibrational mode dynamics can be fitted by Eq. (3.8).
